# Deducing chemical structure from crystallographically determined atomic coordinates

**DOI:** 10.1107/S0108768111024608

**Published:** 2011-07-18

**Authors:** Ian J. Bruno, Gregory P. Shields, Robin Taylor

**Affiliations:** a Cambridge Crystallographic Data Centre, 12 Union Road, Cambridge CB2 1EZ, England; b Taylor Cheminformatics Software, 54 Sherfield Avenue, Rickmansworth, Herts WD31NL, England

**Keywords:** Cambridge Structural Database, structure assignment, *catena* structure, disorder resolution, Bayesian statistics

## Abstract

An improved algorithm has been developed for assigning chemical structures to incoming entries to the Cambridge Structural Database, using only the information available in the deposited CIF. Steps in the algorithm include detection of bonds, selection of polymer unit, resolution of disorder, and assignment of bond types and formal charges. The chief difficulty is posed by the large number of metallo-organic crystal structures that must be processed, given our aspiration that assigned chemical structures should accurately reflect properties such as the oxidation states of metals and redox-active ligands, metal coordination numbers and hapticities, and the aromaticity or otherwise of metal ligands. Other complications arise from disorder, especially when it is symmetry imposed or modelled with the SQUEEZE algorithm. Each assigned structure is accompanied by an estimate of reliability and, where necessary, diagnostic information indicating probable points of error. Although the algorithm was written to aid building of the Cambridge Structural Database, it has the potential to develop into a general-purpose tool for adding chemical information to newly determined crystal structures.

## Abstract 2

An improved algorithm has been written for assigning chemical structures to incoming entries to the Cambridge Structural Database.

## 1. Introduction   

For 45 years the Cambridge Structural Database (CSD; Allen, 2002 ▸) has been maintained by the Cambridge Crystallographic Data Centre (CCDC) as the definitive collection of small-molecule organic and metallo-organic crystal structures. Throughout this time, the CCDC has had the following core aspirations:

(i) that the database should afford comprehensive coverage of published crystal structures in its area of remit;
(ii) that it should achieve high standards of accuracy;
(iii) that it should be accompanied by effective search software.

The focus of this paper is on the important issue of assigning the correct chemical structure (bond types, formal charges *etc.*) to each incoming entry. This task is obviously germane to the second aspiration but also to the third, since most searches of the CSD are substructure searches which cannot give accurate results unless chemical structures are assigned reliably. If every incoming structure to the CSD were accompanied by an accurate, machine-readable chemical diagram provided by the authors, the problem of structure assignment would be largely solved. However, sadly this is far from being the case, nor is there any indication that it will become so in the immediate future.

The CCDC believes that all molecules in the unit cell are important. It would be easy to assume, for example, that an isolated O atom indicates a water molecule whose H atoms have not been located. However, it might be a hydroxonium ion (954 entries in the CSD, Version 5.32 contain H_3_O^+^) or a hydroxide (occurring in 317 entries), which would obviously have implications for the protonation and oxidation states of other, ‘more important’ moieties in the unit cell. As another example, solvate molecules are often poorly resolved, but CCDC editors still assign their structures with care. This is now paying dividends in the field of crystal engineering; because solvates are identified accurately, the CSD can be used to gain insights into factors governing pseudo-polymorphism and co-crystal formation, both of huge commercial importance (Almarsson & Zaworotko, 2004 ▸).

Most published algorithms for chemical-structure assignment from three-dimensional atomic coordinates (Froeyen & Herdewijn, 2005 ▸; Hendlich *et al.*, 1997 ▸; Labute, 2005 ▸; Sayle, 2001 ▸; Zhao *et al.*, 2007 ▸) were intended for use on the Protein Data Bank (PDB; Berman *et al.*, 2002 ▸) and, specifically, on protein-bound ligands. It might appear that, because of their lower precision, PDB structures offer a greater challenge than those from the CSD. For example, H-atom positions are usually available in CSD structures, which is a significant help. However, this advantage is outweighed by the defining problem of CSD chemical-structure assignment, *viz.* the large number of metallo-organic complexes that have to be processed. This single issue makes CSD structure assignment a severe challenge.

**Figure 1:**
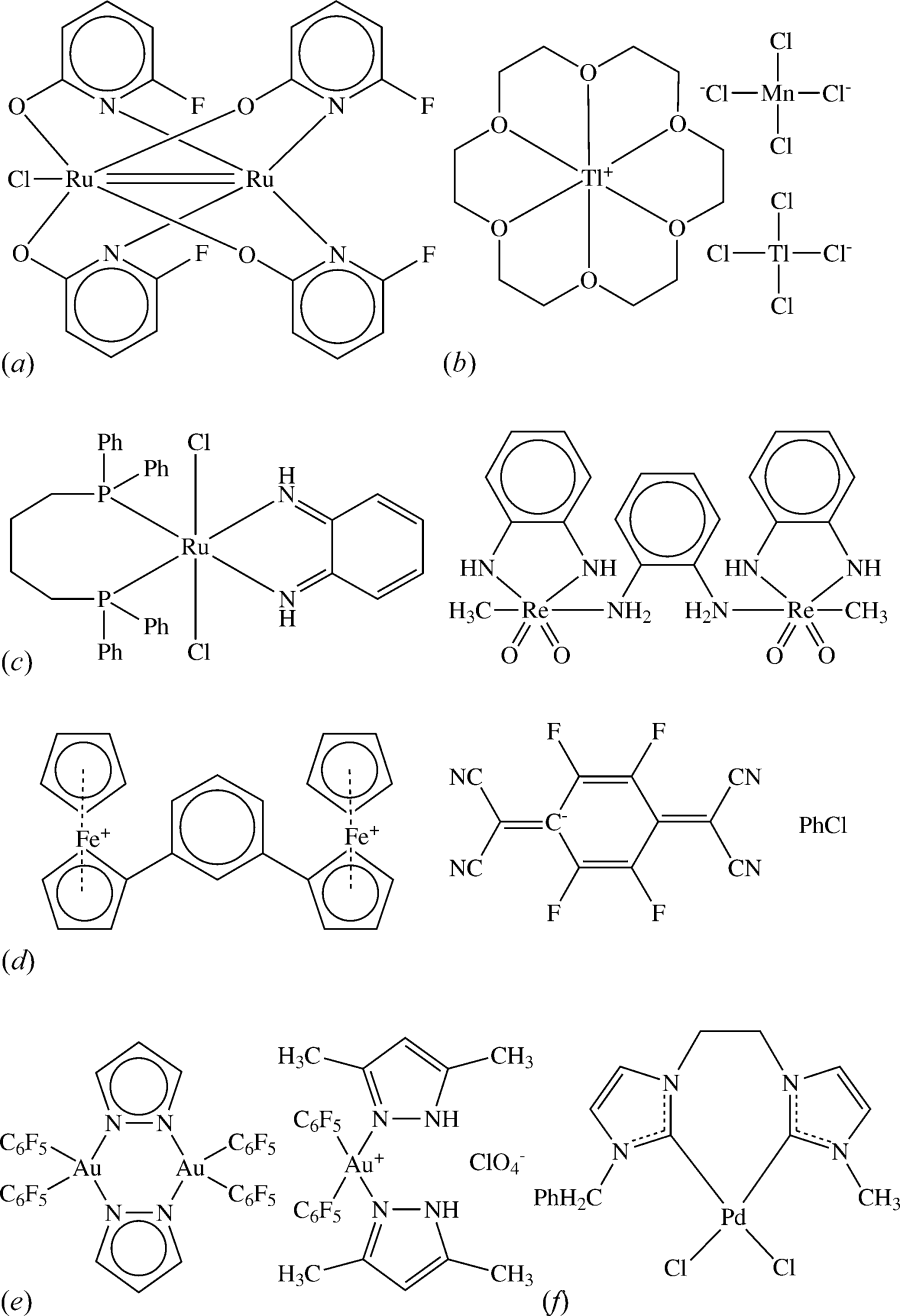
CSD entries (*a*) GEBXOA, (*b*) HEWMOL, where the stoichiometry is 4(C_12_H_24_O_6_Tl^+^), Cl_4_Mn^2−^, 2(Cl_4_Tl^−^), (*c*) YAZZOP (left) and BALTUE (right), (*d*) BAPYEX, where the stoichiometry is C_26_H_22_Fe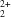, 2(C_12_F_4_N_4_^−^), 2(C_6_H_5_Cl), (*e*) VOMNUH (left) and VOMPAP (right), and (*f*) XONQIB. All structure assignments are as in the CSD, but the metal–metal bond in GEBXOA is actually of half-integral bond order (2.5).

**Figure 2:**
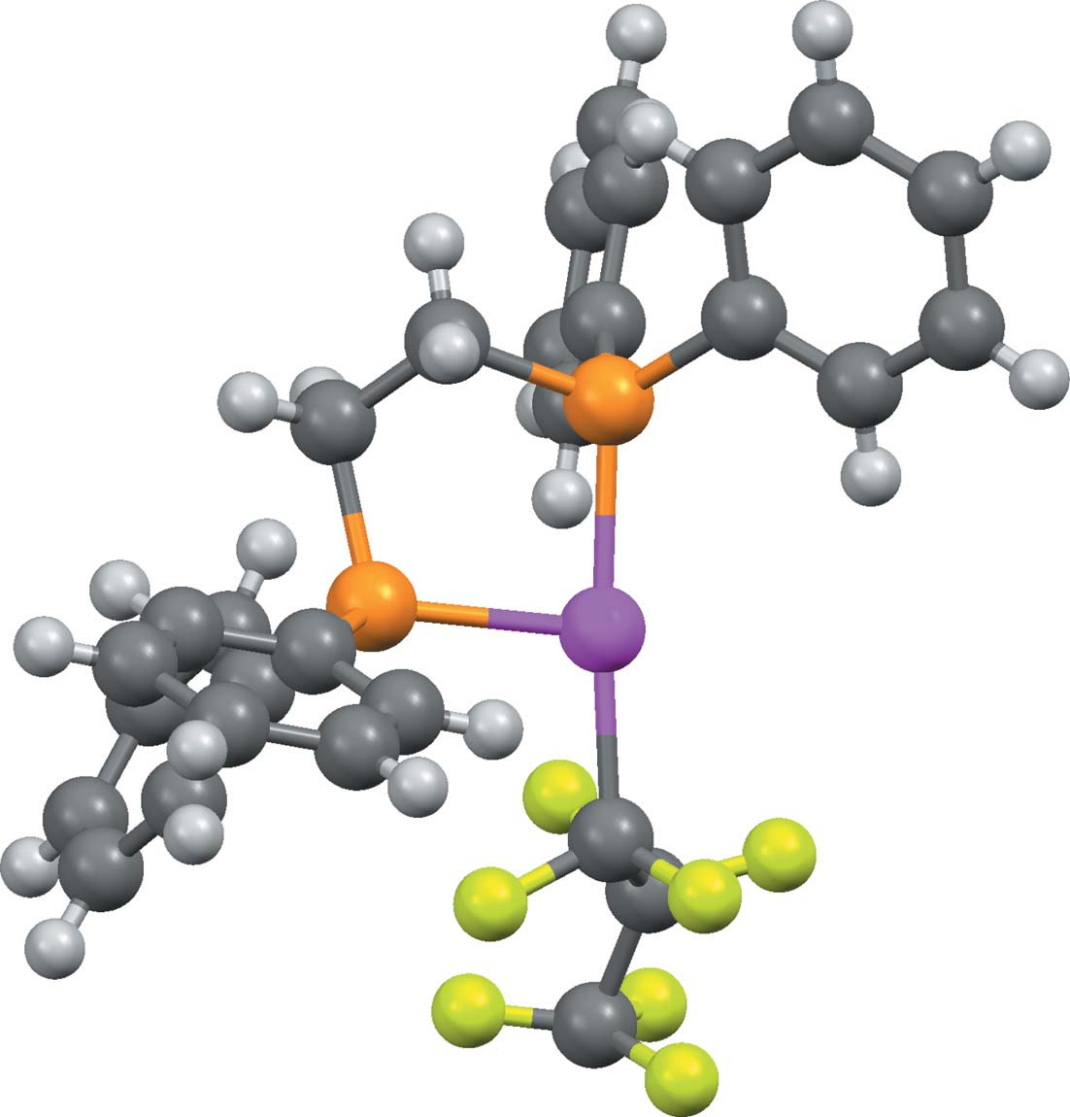
Structure of OFIKOD, (Ph_2_PCH_2_CH_2_PPh_2_)Pt(H)(C_3_F_7_). The coordination around the Pt atom is incomplete because a hydride ligand was not located.

**Figure 3:**
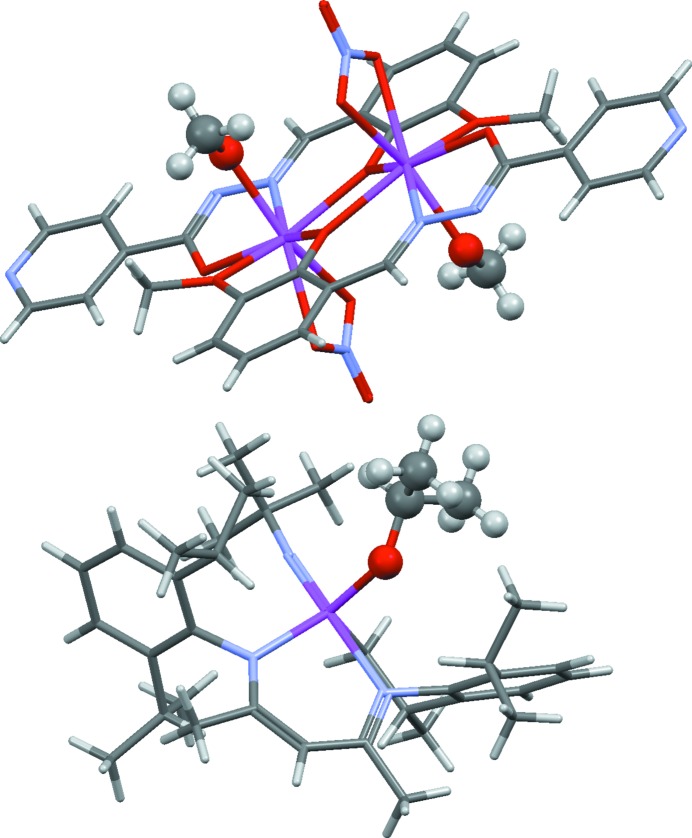
Structures of XOLSIB, Dy_2_(C_14_H_11_N_3_O_3_)_2_(NO_3_)_2_(MeOH)_2_ (top), and VOLSAR, ^*t*^BuN=Nb(O^*i*^Pr)(C_29_H_40_N_2_) (bottom). In both structures, the metal atoms appear to be bonded to alkoxide ligands (shown in ball-and-stick style). In the former, however, the ligand is actually unionized methanol, the alcoholic H atom having not been located.

**Figure 4:**
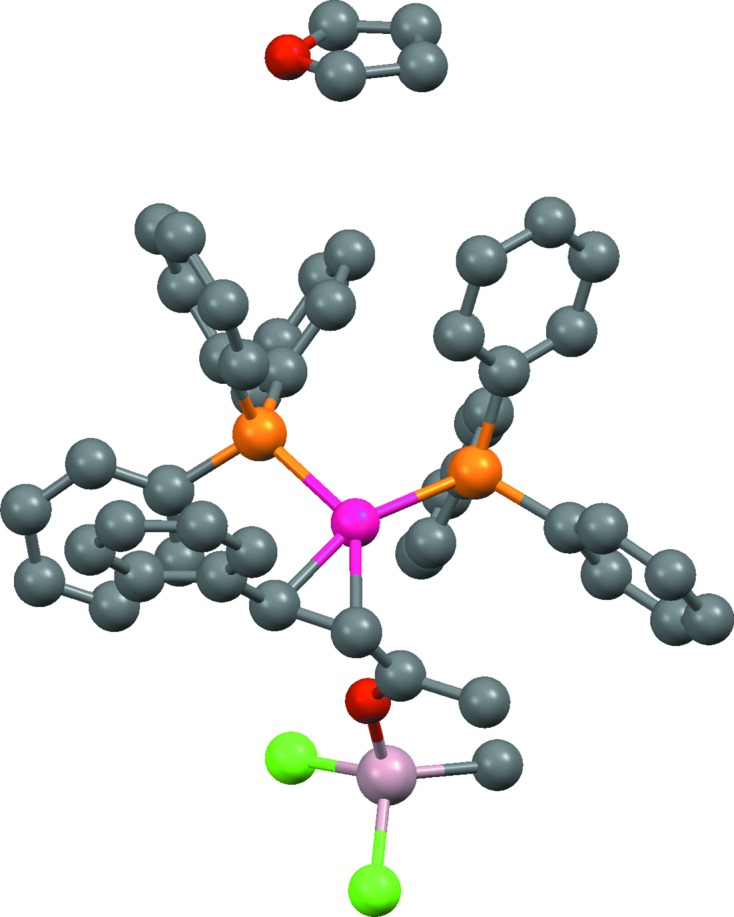
Structure of NOLZOE, [(Ph_3_P)_2_Pd(PhCH=CHC(Me)=O—Al(Me)Cl_2_]·C_4_H_8_O, showing a solvent molecule that is actually tetrahydrofuran despite its apparent near-planar geometry (no H atoms were located).

**Figure 5:**
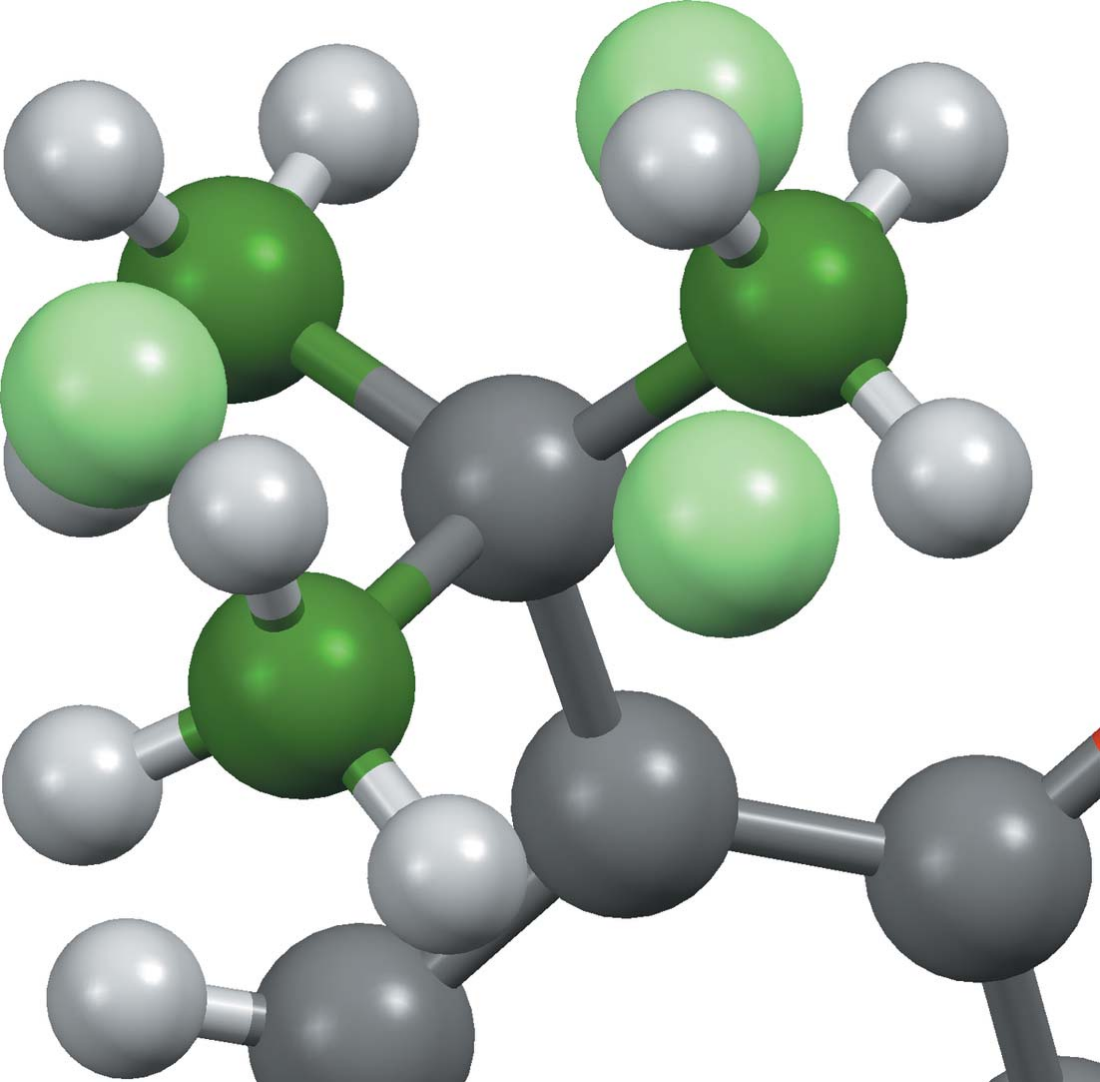
Part of the structure of QEHLOF, showing a twofold disordered *tert*-butyl group. Carbon positions are reported for both configurations of the disordered group but hydrogen positions for only one configuration.

**Figure 6:**
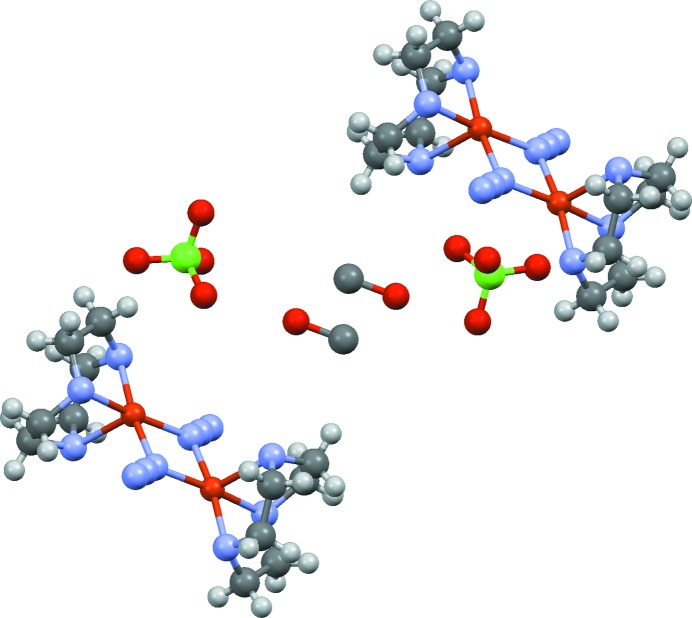
Crystal packing in the structure of DEHMAF, [(1,4,7-triazacyclononane)-Cu-(N_3_)_2_-Cu-(1,4,7-triazacyclononane)][ClO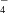]·MeOH. The two C—O fragments at the centre of the figure are related by an inversion centre and could correspond to half-occupancy ethane-1,2-diol or methanol disordered by symmetry over two sites (no solvent H atoms were located). The solvent is actually methanol.

**Figure 7:**
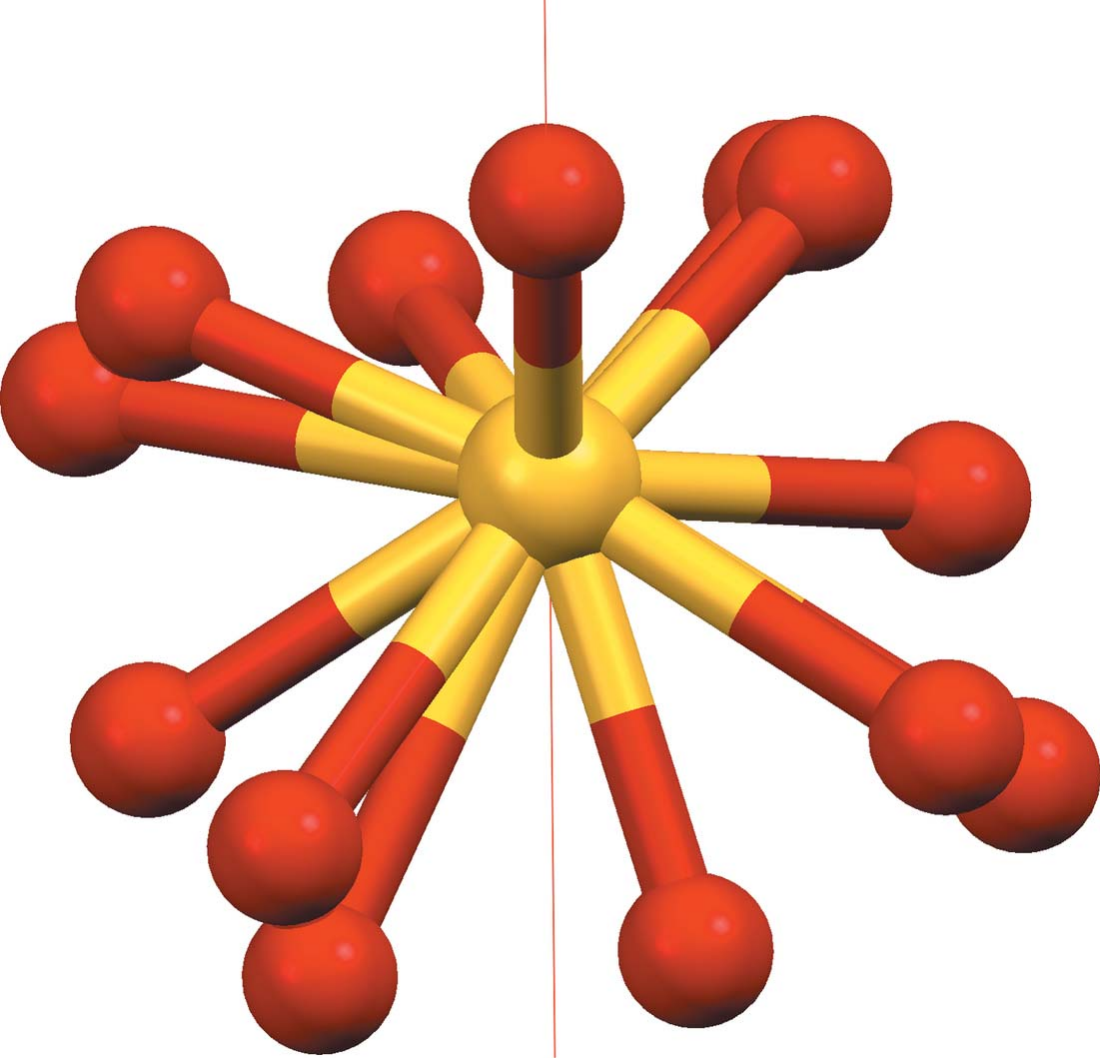
Sulfate ion in AHALEA. The sulfur and one full-occupancy O atom lie on a fourfold axis (shown), resulting in four disordered configurations.

The following is a list of some illustrative problems, based on selected CSD entries (structures are referred to by their CSD reference codes throughout):

(i) In GEBXOA (Fig. 1 ▸ *a*) it is necessary to determine the bond order of the metal–metal bond (it is actually half-integral, *viz.* 2.5).
(ii) In HEWMOL (Fig. 1 ▸ *b*) it is necessary to assign charges to three different metal-containing species, each involving a metal that can exist in more than one oxidation state.
(iii) YAZZOP and BALTUE (Fig. 1 ▸ *c*) contain a redox-active ligand that can act as a neutral benzoquinone-diimine or a charged phenylene-diamide, the implied metal oxidation states varying accordingly. In addition, BALTUE contains metal–oxygen double bonds which could be confused with single bonds to water molecules whose H atoms have not been located.
(iv) In the charge-transfer complex BAPYEX (Fig. 1 ▸ *d*) it is necessary to decide on the charges to be assigned to the organic and metal-containing molecules.
(v) In VOMNUH and VOMPAP (Fig. 1 ▸ *e*) it is necessary to decide whether to represent the pyrazole ligands as aromatic.
(vi) In XONQIB (Fig. 1 ▸ *f*) it is necessary to recognize that the metal is coordinated by carbene ligands.
(vii) In OFIKOD (Fig. 2 ▸) it is necessary to infer the presence of the metal-bound hydride ion, which was not located in the X-ray study.
(viii) XOLSIB and VOLSAR (Fig. 3 ▸) both appear to contain metal-coordinated alkoxides, but the possibility exists that the groups are unionized alcohols with undetermined H-atom positions.
(ix) The solvate molecule in NOLZOE (Fig. 4 ▸) is nearly planar with undetermined H-atom positions, raising the possibility that it might be furan rather than the more common tetrahydrofuran.
(x) In QEHLOF (Fig. 5 ▸) only the major configuration of a disordered assembly has hydrogen sites.
(xi) In DEHMAF (Fig. 6 ▸) it is necessary to determine whether the solvent (whose hydrogen positions are undefined) is half-occupancy ethane-1,2-diol or methanol disordered by symmetry over two sites.
(xii) In AHALEA (Fig. 7 ▸) a sulfate ion is disordered by symmetry about a fourfold axis, the S and one O atom have full occupancy while the other three O atoms are each disordered over four positions with ¼ occupancy.

Frequently, the only reliable way of assigning structure is by manual editing. In particular, when the best representation of a structure is open to interpretation, the CCDC believes that the authors’ view is likely to be the most informed. Unfortunately, if no chemical diagram has been deposited, assimilation of the authors’ opinions can only safely be done by a person reading the relevant journal article. However, the exponential rise in the production of crystal structures makes it unsustainable to look at *every* incoming entry. We have therefore developed a new version of our structure-assignment software, with two aims in mind. First, the algorithm should be capable of correctly inferring the structures of a substantial proportion of incoming entries, including many difficult cases, using only the information in the deposited CIF (Hall *et al.*, 1991 ▸). Second, it should give a good indication of the reliability of any given assignment and, when that reliability is low, should provide pointers towards the likely source(s) of error. Only with meaningful reliability estimates can CCDC editors identify and focus their time on those entries most in need of manual inspection.

Structure assignment involves the following steps: identification of chemical bonds and, where necessary, detection of polymer structures and choice of representative unit; resolution of disorder; assignment of bond types, formal charges and inference of missing H atoms; assessment of reliability. Our methodology for each step is described below, followed by a discussion of results.

## 2. Structure representation conventions and problems   

Apart from the obvious bond types (including aromatic), the CSD also makes use of quadruple bonds for some metal–metal linkages, pi bonds for poly hapto-bound metal ligands, and delocalized bonds. The latter are used for systems such as bidentate acetylacetonato and have the advantage over representations using alternate single and double bonds that they correctly reflect local symmetry. Some metal–metal bonds have non-integral bond orders that cannot be represented in the CSD at present. Recently, quintuple bonds have been reported in some chromium dimers (*e.g.* Nguyen *et al.*, 2005 ▸) and the possibility of even higher order bonds has been discussed (Radius & Breher, 2006 ▸). These bond types are not currently allowed in the CSD, although there should be little difficulty in adding them. There is no mechanism in the CSD for indicating a radical, which makes it impossible to accurately show the bonding in *e.g.* structures involving semiquinone anion radicals.

**Figure 8:**
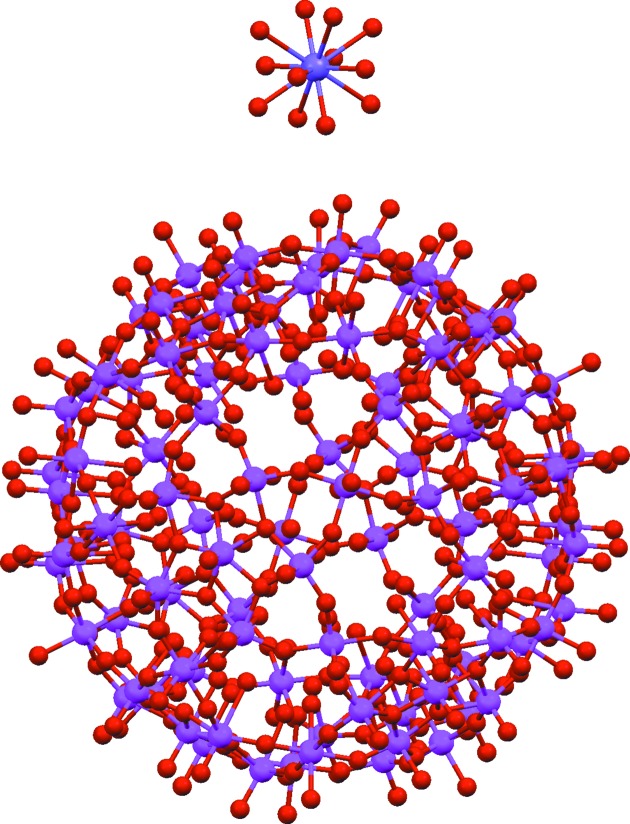
Structure of DIJWEZ, Na(H_2_O)
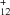
·Cr_30_Mo_72_C_38_H_245_O_384_·120H_2_O (disordered atoms and waters omitted). The ion at the top of the figure is dodecaaqua-sodium, obviously a monocation, leaving the question as to where the balancing negative charge on the keplarate ion should be placed.

Charge representation can be difficult. For example, the Keplerate anion in DIJWEZ (Fig. 8 ▸) has a net charge of −1. How should this be represented? It would be tedious and futile to attempt an accurate representation of the charge distribution by placing integer or partial charges on all or most of the metal and O atoms. Assigning an overall charge to the ion as a whole, rather than on any individual atom, is sensible. However, this approach is problematic if an ion treated in this way contains an atom which any chemist would expect to be formally charged, *e.g.* quaternary nitrogen. The compromise currently used in the CSD is to assign charges only to individual atoms (accepting that the choice of atom is sometimes arbitrary), making the assignment as chemically sensible as possible, and ensuring that the charges on the atoms of each molecule or ion add up to the correct value. Even this is sometimes inadequate, *e.g.* in charge-transfer complexes when it may be hard to quantify the charge on an ion.

## 3. Bond detection and choice of polymer unit   

**Figure 9:**
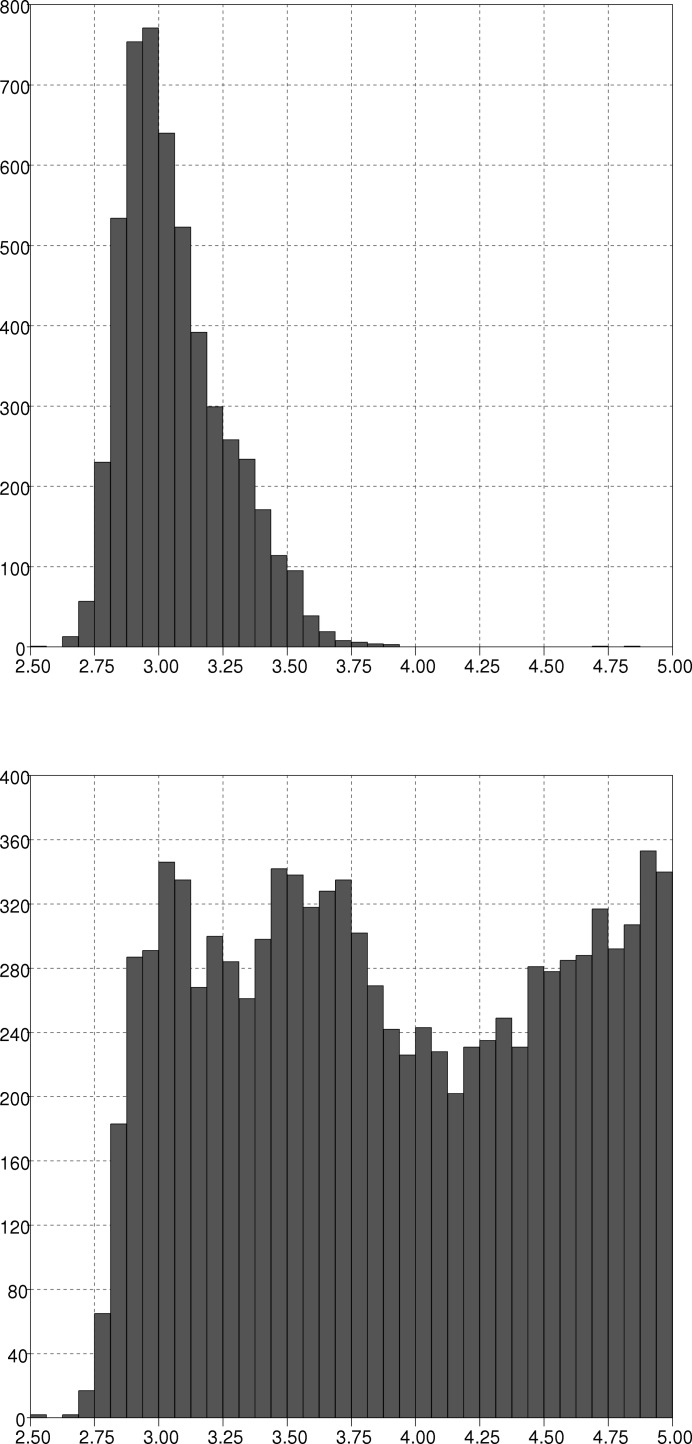
Histogram of (*a*) Ag—Ag bond distances and (*b*) Ag—Ag non-bonded contact distances (intramolecular and intermolecular) in the CSD.

The detection of bonds and symmetry expansion is based on the Unique Molecule Program (Allen *et al.*, 1974 ▸). However, instead of using elemental radii, an upper distance limit for each element–element pair was employed, allowing the finer control of bond-distance limits. For many element pairs, the starting values were the sum of the CCDC covalent radius values (Cambridge Crystallographic Data Centre, 2011 ▸) plus a tolerance of 0.45 Å. Values for bonds between *s*-block and *p*-block elements (*e.g.* Na—O) were based on the *s*-block radii of Kerr (2002 ▸) plus a tolerance of 0.40 Å. A utility program was written for comparing the connectivity calculated with these distances with the connectivity in the CSD. For each element pair, the program produces a list of the lengths of bonds that are (*a*) present in the new connectivity but not in the CSD, and (*b*) *vice versa*. This program was used to validate the distance limits for a subset of *ca* 32 000 entries. Where there were many discrepancies for an element pair, values were manually optimized by inspection of histograms of bonding and non-bonding distances in the CSD and the validation repeated. In a number of cases (*e.g.* Ag—Ag bonds, Fig. 9 ▸) there is substantial overlap between bonding and non-bonding distance distributions, reflecting differing opinions of the authors of the original publications.

**Figure 10:**
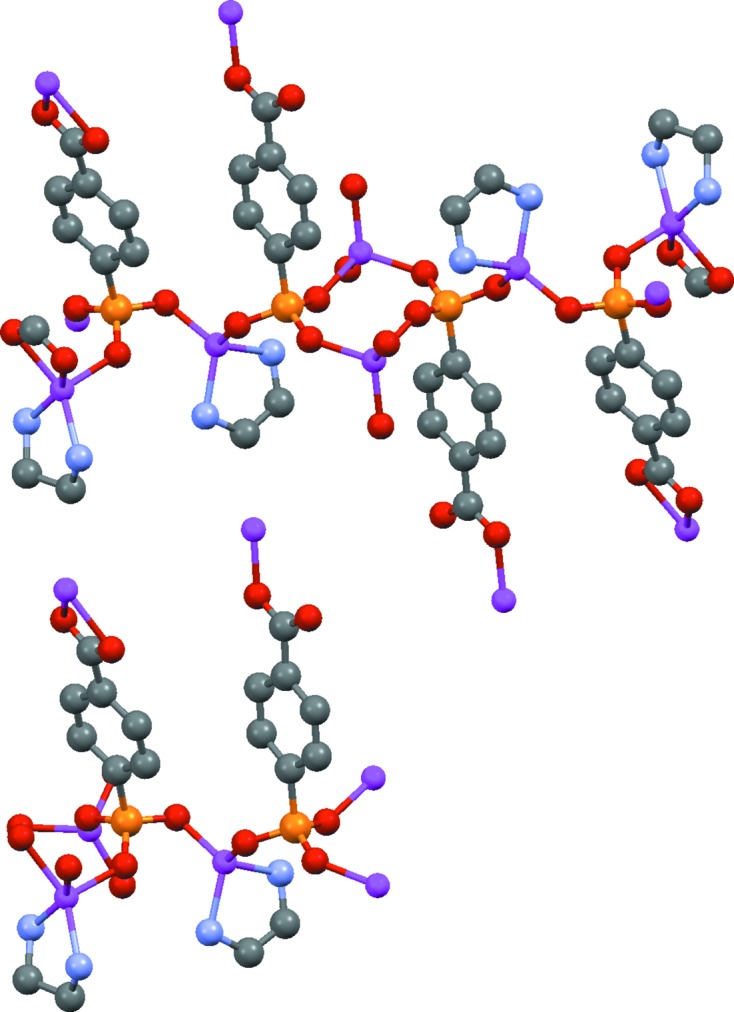
Two possible choices for the polymer unit of XOJWUP, [Zn_3_(C_7_H_4_PO_5_)_2_(NH_2_CH_2_CH_2_NH_2_)_2_]_*n*_ (H atoms omitted for clarity): at the top, the unit found by the algorithm reported herein; at the bottom the smaller unit chosen by CSD editors. Although unnecessarily large, the former arguably gives a clearer picture of the bonding. Some peripheral atoms in both representations are ‘link atoms’, *i.e.* atoms of adjacent units of the polymer.

It is important that the symmetry expansion process leads to a chemically sensible choice of molecular unit which is a multiple of the formula unit. To avoid problems with incomplete ligands in polymeric metallo-organic structures (by CCDC convention, polymeric chains are terminated with metal–metal or metal–ligand bonds where possible), the symmetry expansion proceeds by a number of separate steps. Initially, only bonds between non-metals are considered and used to generate complete symmetry-expanded ligands. Only non-translational operators are applied to the input atoms to ensure that a finite ligand unit is generated. The multiplicity of each ligand and metal atom is then calculated. The ligands and metal atoms (if any) are then symmetry expanded, again only allowing non-translational operators. For each connected unit which results from this expansion, ligand and/or metal atoms are then removed as required to ensure that they are included in the final molecular unit with the correct relative multiplicities (this is only necessary if the molecular unit is polymeric). This procedure generates the largest possible subset of the (non-translationally) symmetry-expanded unit representing a multiple of the formula unit. In some cases it may result in a polymer unit which is larger than the minimum necessary, for example XOJWUP (Fig. 10 ▸). However, the chemistry is arguably made clearer by the inclusion of the extra unit resulting from the application of the inversion operator in the symmetry expansion.

**Figure 11:**
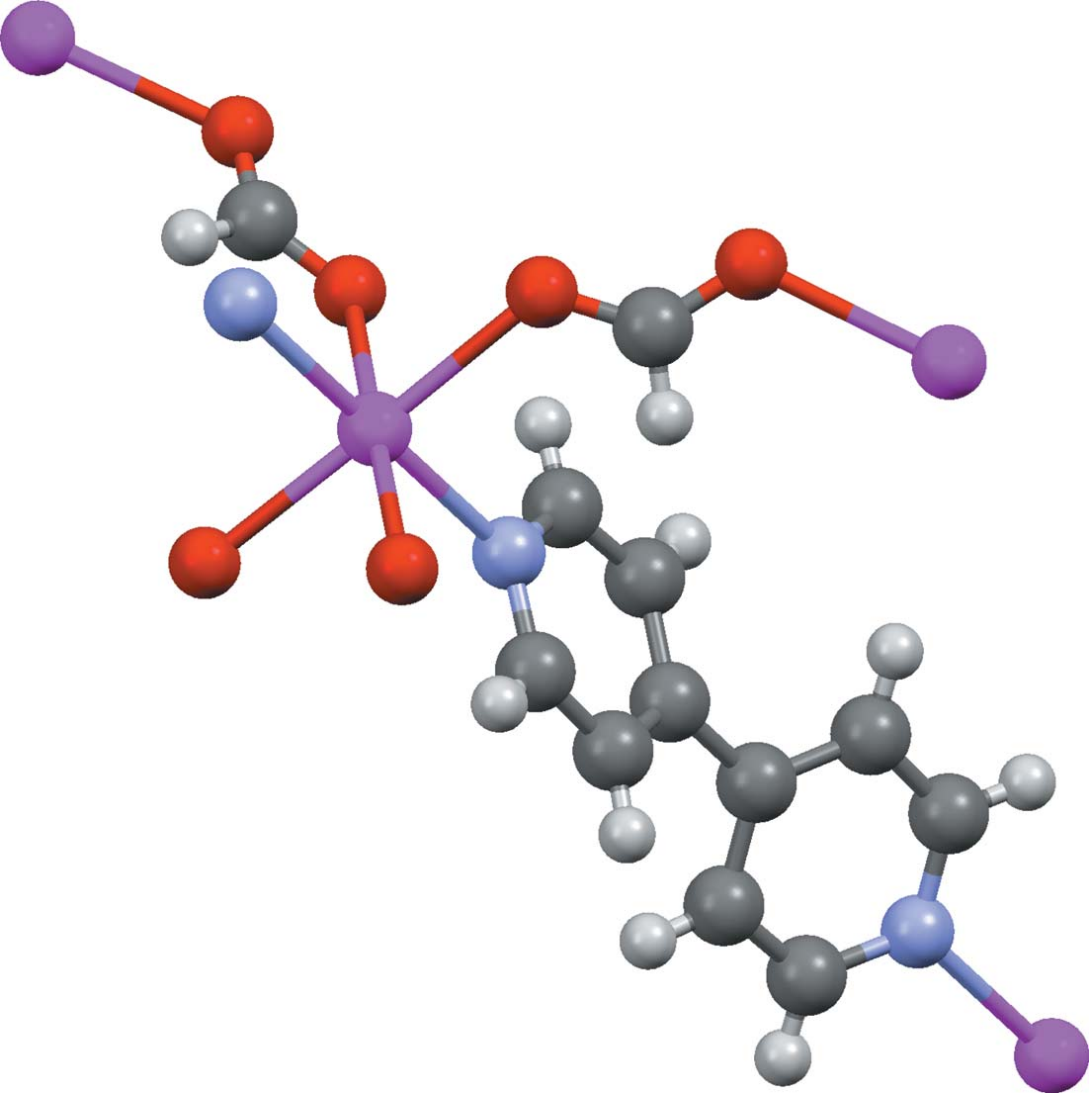
Polymer unit of VOQBUZ, [Cd(C_10_H_8_N_2_)(CHO_2_)_2_]_*n*_. Only one of the two formato ligands required to maintain stoichiometry is generated by initial symmetry expansion using non-translational symmetry operators. The problem is detected, and symmetry expansion repeated, allowing a limited number of translational operators.

If it is not possible to achieve the correct formula unit by removing complete ligands or metal atoms from the symmetry-expanded molecule (*e.g.* in VOQBUZ, Fig. 11 ▸, where only one carboxylate ligand is generated rather than the required two), the symmetry expansion is repeated, allowing a limited number of translational operators, and the removal process repeated. Finally, polymeric ‘link atoms’ are added at any points where the structure can be extended by translational symmetry. However, to avoid polymeric expansion of poorly resolved solvent, polymeric links are not expanded for molecules with less than 2/3 occupancy.

The resulting bonds are subsequently refined to remove commonly occurring short contacts which are not represented in the CSD as bonds, including those listed below.

(i) Agostic contacts between C—H, N—H and O—H groups and metal atoms.
(ii) Contacts between metal atoms and B, Ga, In, C, Si, Ge, Sn, P, As, Sb, S and Se atoms that are bonded to other non-metal atoms in tetrahedral, trigonal-bipyramidal, square-based pyramidal or octahedral coordination geometries.
(iii) Contacts between metal atoms and non-metal atoms (as listed above) that are bonded to other non-metal atoms (other than H or D, as positions may not be reliable) in tetragonal-pyramidal, sawhorse or trigonal-pyramidal geometries, where the angle between the (central atom)–(coordinating-atom centroid) vector and the (central atom)–(metal atom) vector is less than 110°. The intent is to retain a bond between a central, pyramidal non-metal atom and a metal atom only if the metal is positioned on or close to the lone-pair direction (angle of 180°). For example, in a metal-PMe_3_ complex the angle is between the vector from P to the centroid of the three C atoms and the vector from P to the metal.
(iv) Contacts between metal atoms and B, C and N atoms bonded to non-metal atoms (other than H or D) in a trigonal-planar geometry, where the angle between the normal to the plane defined by the non-metal atoms and the central (B, C or N atom)–(metal atom) vector is in the range 60–120° (this removes spurious bonds between C atoms of carboxylate ligands and metal atoms).

The algorithm described above is currently used in the CCDC program *Mercury* (Macrae *et al.*, 2006 ▸, 2008 ▸).

## 4. Resolution of disorder   

Disorder assembly and group information may be given explicitly in the CIF using the _atom_site_disorder_assembly and _atom_site_disorder_group data items. Alternatively, it may possibly be deduced from site occupancies (_atom_site_occupancy). We have developed improved algorithms for resolving disorder, making use of all these data items.

Each independently disordered part of the structure constitutes an assembly. An assembly can have two or more groups of disordered atom sites associated with it, atoms in the same disorder group representing a particular configuration of the disorder assembly. For example, a molecule with three independent CF_3_ groups, each disordered over two configurations, would be represented with three disorder assemblies, each containing two groups of three partial-occupancy F atoms. Validation of the assemblies and groups given in the CIF is the first step in disorder analysis. Assembly information is discarded if there are any assemblies containing only a single atom site (in practice, this usually happens when authors mistakenly put each disordered atom site in its own assembly), or if the sum of group occupancies in an assembly is greater than 1. Assembly and group information is discarded for atoms with full or zero occupancy. If different assemblies contain sites for the same atoms (*i.e.* authors have put different assembly flags in the CIF when they should have put different group flags), the assemblies are merged together provided the sum of the resulting group occupancies is 1. Otherwise, the assembly and group information is discarded.

The next step is to assign atoms with partial occupancy to disorder assemblies and groups where these are not given in the CIF or have been discarded as invalid. Assigning the atoms to a disorder assembly can be straightforward, but assigning to each one of those atoms its corresponding disorder group is more difficult. For each assembly, the distinct occupancy factors (those which differ by more than one standard uncertainty) of atoms in the assembly are searched for pairs or triplets that sum to 1, *e.g.* {0.44, 0.56} or {0.454, 0.247, 0.299}, representing two and threefold disorder, respectively. A search is also made for pairs of distinct occupancy factors which sum to ½ (since partial occupancy molecules sometimes have their total occupancy fixed at ½). Where the pattern of occupancy factors is unambiguous, the atoms are then divided into disorder groups according to these occupancy factors; for example, it would be possible if the pairs were {0.44, 0.56} but not if they were {0.5, 0.5}. Half-occupancy atoms are considered in a later stage of the disorder analysis, after symmetry expansion.

Molecular connectivity is generated without symmetry expansion, excluding bonds between atoms in the same disorder assembly but different groups. For each disorder assembly, a graph match is performed between the primary disorder group (group with largest occupancy) and the lower-occupancy groups. If they match, the analysis is considered to be successful for that assembly and is retained. Otherwise, the group and assembly information is discarded.

The molecular connectivity is then generated with symmetry expansion (as described above), excluding bonds between different disorder groups (where these have been retained) as above. Disorder assembly and group information is copied to symmetry-generated copies of partially occupied atoms. The crystal connectivity is then divided into discrete molecules. For each molecule, the maximum and minimum occupancies are calculated. If the minimum occupancy is 1, the molecule is not disordered. If the maximum occupancy is not equal to 1/4, 1/3, 1/2, 2/3 or 3/4, the molecule is assumed to have overall partial occupancy; otherwise, the total occupancy is assumed to be 1. The atoms in the molecule are divided into 1/2, 1/3, 1/4 and full occupancy atoms (relative to the maximum occupancy). Atoms with 2/3 occupancy are counted as two atoms with 1/3 occupancy since they can participate in two disorder groups in an assembly with three groups. An example of this is UKEHEX, which has three oxygen atoms O12, O13, O14 with 2/3 occupancy disordered over three configurations {(O12, O13), (O13, O14), (O12, O14)}. Similarly, atoms with 3/4 occupancy are counted as three atoms with 1/4 occupancy. Atoms with 1/2, 1/3 and 1/4 occupancy are then analysed separately as twofold, threefold and fourfold disordered assemblies.

The analysis proceeds by dividing the partial occupancy 1/*n* atoms first into assemblies and then, for each assembly, into *n* groups. Partial occupancy atoms are placed in the same assembly if they are bonded to any other atom in the same assembly directly or *via* another (full occupancy) atom. If this results in an assembly that could not be partitioned equally into *n* groups (such that each group contains the same number of atoms of each element), assemblies with the same elemental composition are merged to achieve this. Each assembly is then partitioned into *n* groups. There are potentially a large number of possible combinations (for example, there are 924 ways of partitioning 12 disordered C atoms into two groups). A scoring function has been developed to find the partition which gives the best bonding distances. To reduce the number of combinations which need to be considered, an exclusion matrix is derived representing atoms which cannot occur in the same group because of unrealistically short bonding distances. H atoms are considered separately once the best heavy atom combination has been found, to further reduce the combinatorial complexity. For each disorder assembly, a graph match is performed between the first and other disorder groups and, if they match, the analysis has succeeded for the assembly. If analysis of 1/2 occupancy atoms does not succeed, each 1/2 occupancy atom is considered as two atoms with 1/4 occupancy and the analysis repeated. Conversely, if analysis of the 1/4 occupancy atoms fails, the analysis is repeated treating them as 1/2 occupancy atoms in a molecule of 1/2 the maximum occupancy.

If there are no partial occupancy atoms remaining un­assigned to disorder assemblies and groups, then the analysis has succeeded. This is not always the outcome. A common reason for failure is that disorder groups in an assembly do not match. Examples include QEHLOF, where only the major configuration has hydrogen sites, and BESDAF01, where a spurious F—F bond is detected in one disorder group. More fundamentally, mixed-element disorder (where the chemistry of the disordered groups in a single assembly differs) cannot be analysed using this approach.

Disorder cannot be represented fully in the CSD at present and the current approach is to ‘suppress’ minor occupancy atoms. Accordingly, after disorder analysis is completed, analysed atoms which are not in a major occupancy group are suppressed, as are any partial occupancy atoms not analysed with occupancy less than 1/2.

## 5. Bond type and charge assignment   

### 5.1. Introduction   

Most structure-assignment algorithms deduce bond types and formal atomic charges by analysing molecular geometry; for example, if a C—C bond is 1.34 Å in length, it is probably double or aromatic. Combining this type of logic with valency considerations and aromaticity perception often leads to correct inference of structures. However, a drawback of the approach is illustrated by a quote from Labute (2005 ▸), discussing the results of applying his algorithm to a PDB ligand: ‘*The claimed ligand of 2trm is benzamidine, and our method detects a cyclohexene ring instead of a benzene ring because the ring C atoms are highly nonplanar … In our opinion a perception method should not perceive the 2trm ligand as benzamidine – perceiving benzene in this situation implies that cyclohexene could not be perceived correctly*’.

**Figure 12:**
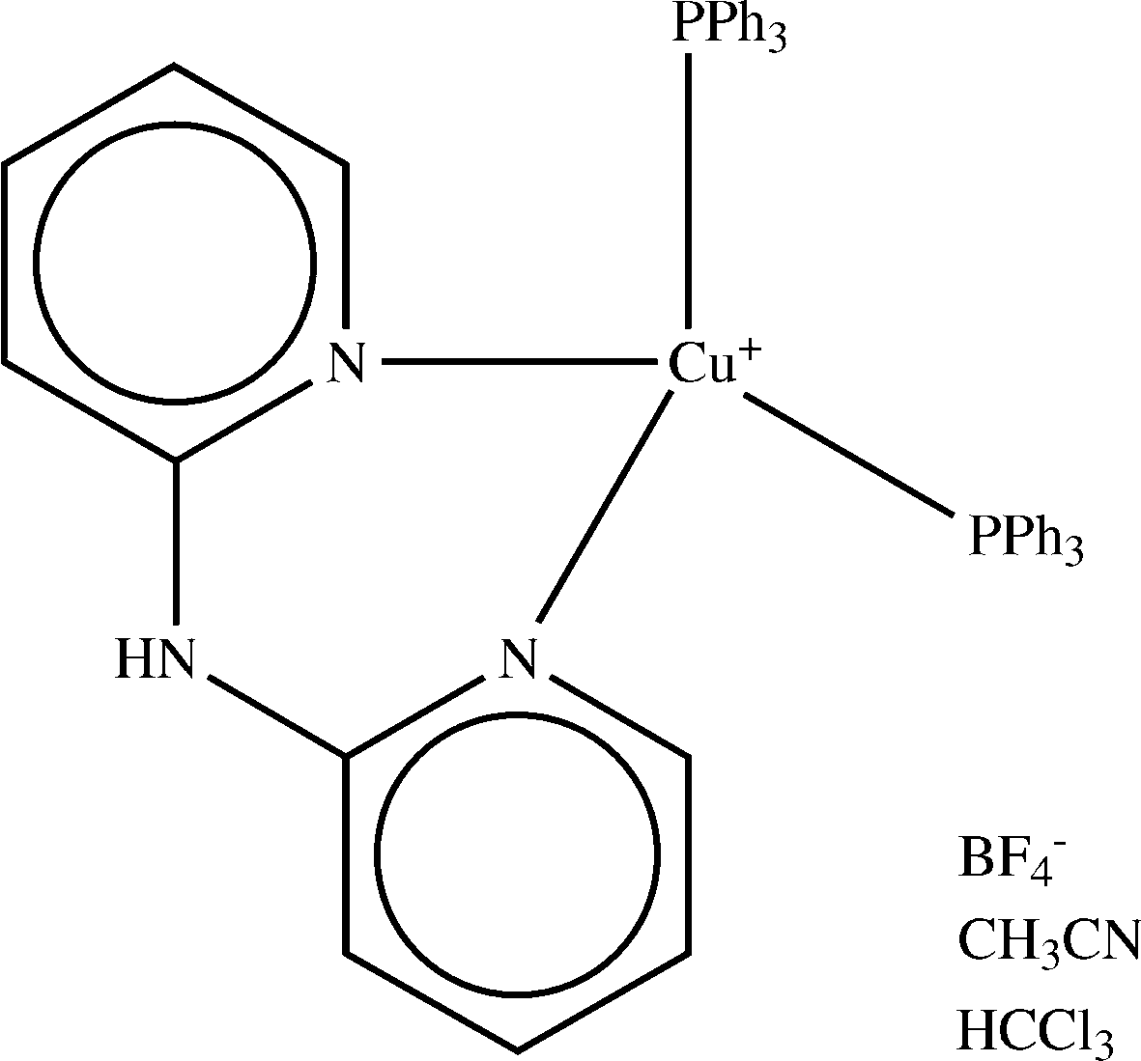
CSD entry MIJFOA. All H atoms were located except those of the acetonitrile molecule, the geometry of which is very bent (C—C—N angle of 135.3°).

While the argument is cogent, the fact remains that benzamidines are relatively common ligands in the PDB whereas their cyclohexene analogues are not. Geometry considerations notwithstanding, the *a priori* expectation is that the ligand is benzamidine. Similar situations often occur in the CSD. For example, the C—C—N bond angle in the acetonitrile solvate molecule of MIJFOA (Fig. 12 ▸) is 135.3°, much closer to that expected for CH_2_=CHNH_2_, CH_3_CH_2_NH_2_ or CH_3_CH_2_NH_3_^+^ than that expected in CH_3_C N (all are theoretically possible since the solvate H atoms were not located). However, while the geometry suggests one of the amine or ammonium forms, acetonitrile is ubiquitous in the CSD whereas the alternatives are very rare.

The problem, therefore, is that structure assignments suggested by geometry considerations alone may conflict with prior expectations based on the frequencies with which different moieties occur in the CSD. It seems desirable to base assignments on both strands of evidence, which can be done by use of Bayes’ formula
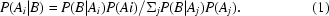

*P*(*A_i_*) is the prior probability of a particular chemical fragment (*e.g.* the above CCN solvate molecule) having a particular structure *A_i_* (*e.g.* CH_3_C N) based on the frequency with which that structure is seen in the CSD. *P*(*B*|*A_i_*) is the conditional probability (likelihood) of observing a particular geometry (*B*) in the fragment (*e.g.* C—C—N angle of 135 ± 5°) if its structure is *A_i_*. The sum in the denominator is over all possible structure assignments, *A_j_*. The result, *P*(*A_i_*|*B*), is the posterior probability that the correct assignment is *A_i_*.

The Bayesian approach allows us to use the knowledge already in the CSD to help assign structures to new entries. It cannot be used on its own, however, because the information in the CSD is limited. We therefore combine it with a previously published, more conventional geometry-based algorithm (henceforth, the *Mogul algorithm*) which was developed for use with the Mogul knowledge-base (Bruno *et al.*, 2004 ▸), *i.e.* we use the Bayesian method where we can and the Mogul algorithm to complete the assignment. Bayesian probabilities also have a crucial role in assessing structure-assignment reliability.

### 5.2. Untyped fragments   

We define an *untyped fragment* (henceforth, UF) as a connected group of atoms that has correctly identified bonds but no bond types or atom charges assigned, and with all H atoms removed. UFs may range in size from a single atom to a complete molecule. For example, the molecules CH_3_C N and CH_3_CH_2_NH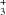 both correspond to the same *complete-molecule UF*, *viz.* C~C~N, where ~ indicates a bond of unspecified type. A UF has one or more *bond-type option(s)*, these being the bond-type assignments that are found in the CSD for that fragment. The complete-molecule C~C~N fragment has two bond-type options, *viz.* C—C—N and C—C N, no other assignment being present in the CSD (ignoring one obvious error).

Each bond-type option is associated with one or more *atom-property option(s)*, these being the various hydrogen counts and atom charges that have been observed in the CSD for the combination of UF and bond-type option under consideration. Table 1 ▸ summarizes all bond-type and atom-property options for C~C~N, along with the number of times each has been observed in the CSD (Version 5.28). The data in the table permit the calculation of frequency-based prior probabilities – the *P*(*A_j_*) in (1). *A priori*, if all H atoms are missing, the probability that a C~C~N molecule in an incoming CSD structure is acetonitrile is 0.991 (= 5102/[5102 + 40 + 4], see Table 1 ▸). However, this probability might be modified by the presence of H atoms. For example, if the incoming structure contains the connected unit CH_3_CH_2_NH_2_ (with those H atoms present in the atom-coordinate list), the bond-type option C—C N becomes impossible and the only remaining option is C—C—N. There is then an *a priori* probability of 0.909 (= 40/[40 + 4]) that the molecule is CH_3_CH_2_NH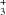 (with one H atom unlocated), the alternative being that it is CH_3_CH_2_NH_2_. The H-atom presence in the incoming structure is always used in this way to eliminate impossible options before Bayesian probability calculations on those that remain.

### 5.3. Geometry tests   

For any UF, we can manually define (or, in certain cases, the software can define automatically) one or more geometry tests to help discriminate between the various bond-type and atom-property options. For example, three tests are defined for the UF discussed above, on the CCN angle and the CC and CN distances. The mean and standard deviation of these parameters for each bond-type/atom-property combination are computed from the CSD. If there are insufficient data for a standard deviation to be estimated reliably (by default, < 10 observations), it is set to 0.04 Å, 4 or 10° for distance, valence-angle or torsion-angle parameters. The resulting statistics for C~C~N are given in the last six rows of Table 1 ▸; for example, the table shows that the average CCN angle in CH_3_CH_2_NH_3_^+^ is 113.8° with a standard deviation of 6.2°.

These data are used to compute likelihoods for use in Bayes’ formula. For example, the CCN angle in MIJFOA is 135.3°. For the structure assignment *A_j_* = CH_3_CH_2_NH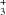, this corresponds to a standardized *z* value of (135.3 − 113.8)/6.2 = 3.47, where standardized *z* is (value − mean)/(standard deviation). The likelihood, *P*(*B*|*A_j_*), of obtaining this value (*B*) within a reasonable tolerance, given the hypothesized structure assignment (*A_j_*), is calculated as
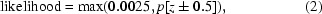
where *p*[*z* ± 0.5] is the area under the normal curve between (*z* − 0.5) and (z + 0.5). Setting the minimum possible likelihood to 0.0025 is an empirical correction to allow for the fact that many geometry-parameter distributions have longer tails than a normal distribution, often with gross outliers. If more than one geometrical test has been defined, the individual likelihoods from each are combined by multiplication to give a single, overall value.

### 5.4. Frequency *versus* geometry weighting of probabilities   

If no geometry tests have been defined for a given UF, all likelihoods – the *P*(*B*|*A_j_*) in (1) – are assumed equal, resulting in probabilities that are based only on frequencies of occurrence. If there is at least one geometry test, the probability can still be biased towards the frequency evidence or, conversely, the geometrical evidence, as follows. Let *P_B_* = *P*(*A_i_*|*B*), the Bayesian probability of structure assignment *A_i_* calculated from (1) using prior probabilities *P*(*A_j_*) derived from the CSD and likelihoods *P*(*B*|*A_j_*) computed from the geometrical test(s). Let *P_f_* be the value that (1) would give if all *P*(*B*|*A_j_*) were set equal, *i.e.* if it were assumed that all structure assignments were equally likely on geometric grounds. Let *P_g_* be the value that would be obtained by setting all *P*(*A_j_*) equal, *i.e.* if it were assumed that all assignments occurred with equal frequency in the CSD. A biased probability can now be calculated as
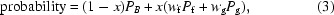
where *x* is a user-specified value between 0 and 1 (the larger the value, the greater the bias), and *w*_f_ = 1, *w*_g_ = 0 for biasing towards frequency or *vice versa* for biasing towards geometry.

### 5.5. Untyped-fragment data files   

The algorithm uses five different categories of UFs: complete molecules; complete ligands; substructures; individual bonds; individual atoms. Data files containing frequency and geometry data were collated from the CSD (version 5.28) for all five categories, as described below. Throughout this section, *non-metal* means any non-metallic element except hydrogen.

#### 5.5.1. Complete molecules   

All complete-molecule UFs that occur at least 100 times in the CSD were included (for example, the complete-molecule fragment C~C~N, see above). Over 200 hand-selected less common UFs were also incorporated, *e.g.* MnBr_4_, to capture the fact that it is always di-anionic. For each fragment, frequency data analogous to that shown in Table 1 ▸ were compiled for all bond-type and atom-property options found in the CSD. For some UFs, where two or more equivalent but equally valid representations are possible, one was given ‘preferred’ status to help promote consistency (this was done by setting the frequencies of the undesired options to zero). When it was deemed worthwhile, geometry tests were defined and the relevant distribution means and standard deviations calculated and added to the data file. The tests were defined manually because the nature of the test was fragment-dependent. For example, to help distinguish pyridine from pyridinium, we defined a test on the C—N—C valence angle, which tends to open out on protonation. Defining the tests is time consuming but, once done, it is easy to update the geometry statistics (and, of course, the frequency data) as the CSD grows.

#### 5.5.2. Complete ligands   

Complete-ligand UFs capture information on metal-bound ligands, *e.g.* there is a cyclic ~C~C~C~C~O~ fragment representing furan, tetrahydrofuran, *etc*. The metal atoms are included in the UF definition so that, for example, monodentate and bidentate acetato ligands correspond to different UFs. However, the type of metal is ignored, *e.g.* an iron-bound acetate would be assigned to the same UF as a nickel-bound acetate with the same coordination mode. Bonds between ligand and metal were taken into account when bond-type options were collated (so a singly bonded ligand–metal was considered a different bond-type option than double-bonded ligand = metal). Charges on metal atoms were ignored when atom-property options were collated since assignment of charges to metals is often somewhat arbitrary in the CSD (see §2). Data were compiled for all complete-ligand UFs occurring at least 200 times in the CSD. Geometry tests, preferred bond-type options *etc.* were defined where necessary as described in the preceding paragraph.

#### 5.5.3. Substructures   

A small number of UFs were manually defined from substructures in which all bond types were set to *any* and no H atoms were included. For example, one such substructure was a generalized description of an (possibly substituted) imidazol-2-ylidene carbene ligand. Any group of atoms in the CSD matching this substructure was deemed to belong to the UF defined by the substructure. Since molecules in the CSD containing this fragment are almost invariably coded with single bonds between the metal and the carbene carbon, use of this UF enables us to avoid the error of assigning a metal–carbon double bond. However, it is difficult to draw substructures that capture only the intended chemical systems. Also, problems were caused by overlapping substructure matches. As a consequence, we made very limited use of this category of UF.

#### 5.5.4. Individual bonds   

Individual-bond UFs comprise just two bonded atoms, *X*~*Y*. They are used primarily to assess the likelihood of multiple bonding involving metals, *e.g.* the Mo~Mo UF has four bond-type options because single, double, triple and quadruple bonds are all possible for this element pair. UFs were created for all *XY* pairs that exist in the CSD. Any *XY* pair involving two metal atoms gave rise to only one UF, *e.g.* all bonds in the CSD between iron and ruthenium were assigned to the same UF. Bonds involving two non-metal atoms were divided into several UFs, depending on whether or not the bond was cyclic and the number of metal and non-metal atoms to which *X* and *Y* were bonded. Bonds between a metal (*X*) and a non-metal (*Y*) were subdivided on the number of metal and non-metal atoms to which *Y* was bound. Bond-type and atom-property options were enumerated for each of the resulting UFs (charges on metal atoms were ignored) and the mean and standard deviation of the *XY* distance computed for use in geometry tests.

#### 5.5.5. Individual atoms   

An individual-atom UF comprises one non-metal atom and its immediate environment (*i.e.* bound neighbours). Specifically, atoms are assigned to the same individual-atom UF if identical in the following respects: atomic number; number of bound metal atoms; number of bound non-metal atoms; atomic numbers, non-metal coordination numbers and metal coordination status (yes or no) of bound non-metal atoms; cyclicities of bonds to bound non-metal atoms. Effectively, this is just a fine-grained atom-typing scheme, but it is convenient to refer to each atom type as an *individual-atom UF* for consistency with the rest of the algorithm. UFs in this category are not used in bond-type assignment but have an important role in charge assignment and in assessing assignment reliability. For example, if an unusual charge has been placed on an atom, it will be detected by the relevant individual-atom UF as being of low-probability, resulting in a warning to the user. All individual-atom UFs occurring at least once in the CSD were included in the data file. For each, all bond-type and atom-property options found to exist in the CSD were collated and the appropriate frequency data computed. A single geometry test was added automatically for all UFs in which the atom was bound to one, two or three non-metals, the test being on bond length, bond angle, and sum of bond angles, respectively.

### 5.6. Metal oxidation-state frequencies   

One of the most important ways of assessing the reliability of a metallo-organic structure assignment is to calculate the metal oxidation state(s) that the structure assignment implies and estimate how probable it is (or they are). In order to do this, we needed a table of observed frequencies of occurrence of metal oxidation states. Since oxidation state is not currently a searchable field in the CSD, we inferred the data indirectly by parsing CSD compound names. Each name was searched for regular expressions that unambiguously indicated the presence of metals, including less-common variants such as *argent(ate|a)*. We also searched for expressions indicating oxidation state, such as (0) or (vii). When (and only when) a compound name was found to contain just one metal and one oxidation state, the count of that metal in that oxidation state was incremented by one, resulting ultimately in the desired table of oxidation-state frequencies.

### 5.7. Bond-type and atom-charge assignment, overall procedure   

The assignment of bond types and atom charges, and inference of missing H atoms, will be described using MIJFOA (Fig. 12 ▸) as an example. The overall procedure is as follows:

(i) Identify untyped fragments present in the structure and select those to be used for structure assignment. Calculate the probabilities of bond-type and atom-property options of the selected untyped fragments.
(ii) Using bond-type option probabilities, assign most probable bond types (if first time through the procedure) or most probable not yet tried (on subsequent iterations). Use Mogul algorithm to assign types to any bonds for which no untyped-fragment information is available.
(iii) Assign most probable atom properties that can be found, given the bond types assigned in step (ii)[Other l4li2].
(iv) Assess reliability of assignment.
(v) If assignment more reliable than any found previously, store it as the best assignment found so far.
(vi) If best assignment found so far is good enough, go to step (viii)[Other l4li8].
(vii) If there are further bond-type option combinations to try, go to step (ii)[Other l4li2].
(viii) Accept best assignment.

### 5.8. Bond type assignment   

The first action is to identify all UFs present in the structure under consideration that are also present in the data files described above. For MIJFOA these are:

(i) three complete-molecule UFs;
(ii) three complete-ligand UFs, two of which are identical;
(iii) individual-bond UFs for all bonds in the structure not involving hydrogen;
(iv) individual-atom UFs for all non-metal atoms in the structure, except H atoms.

It is unusual to find so many complete-molecule UFs; in this case it is because MIJFOA contains three very common molecules or ions (acetonitrile, chloroform and tetrafluoroborate). It is less unusual to find, as here, that all the metal ligands are ‘recognized’.

For each bond in the structure (excluding bonds to hydrogen), the algorithm then decides which UF (if any) is the best source of information about the likely bond type of that bond. Complete-molecule UFs are regarded as the most reliable source, followed by individual-bond UFs (but only for metal–metal bonds and some types of metal–non-metal bonds), complete-ligand UFs and finally substructure UFs (larger substructures preferred over smaller if they overlap). In MIJFOA, for example, the algorithm uses the complete-molecule C~C~N UF to guide bond-type assignment of the bonds in the CCN molecule. It may be (though not in MIJFOA) that some bonds have no UF chosen for them, in which case the types of those bonds will eventually be set by the Mogul algorithm.

At this point, a subset of the UFs has been chosen for use in bond-type assignment. In MIJFOA, this includes all the complete-molecule and complete-ligands UFs but nothing else. The probabilities of all bond-type options of these surviving UFs are calculated. Unbiased Bayesian probabilities are used except for individual-bond UFs, where experience showed that a bias towards geometric data is desirable (referring to §5.4, we use *x* = 0.6, *w*_f_ = 0, *w*_g_ = 1).

The bond-type options are sorted in descending order of probability. Extremely improbable options (*p* < 0.05) are rejected. In the case of MIJFOA, the outcome is unequivocal. Three bond-type options have probabilities of 1. These correspond to the tetrafluoroborate ion, chloroform molecule and bispyridylamine ligand. The result indicates that only one bond-type assignment is present in the CSD for each of the relevant UFs. (In the case of the bispyridylamine ligand, other options are, of course, theoretically possible, *e.g.* the rings could be saturated, since H atoms might be missing from the structure – but as none of these alternatives has been seen in the CSD they are unlikely. If the correct option were indeed one of these novel alternatives, the error would probably be revealed at the reliability-assessment step, described later.) The most likely bond-type option for the C_18_P complete-ligand UF has a probability of slightly less than 1, but sufficiently high that all other options are rejected as too improbable. There are several different ligands in the CSD that correspond to this UF, *i.e.* triphenylphosphine, tricyclohexylphosphine, bis(cyclohexyl)phenylphosphine, *etc*. However, the frequency data combined with the geometry tests associated with this UF leave little room for doubt that the correct assignment is triphenylphosphine. Finally, the C_2_N UF correctly suggests that the molecule is most likely to be acetonitrile. Despite the very bent C—C—N angle, the high frequency with which CH_3_C N occurs in the CSD dominates the Bayesian probability.

When results are not as clear cut, an iterative process is followed. At the start of cycle 1, the most probable bond-type option for each of the UFs in use is chosen. Bond types are assigned accordingly. If any bonds remain unassigned, their types are set by the Mogul algorithm. Atom properties are set (see §5.9) and the reliability of the resulting assignment assessed (§6). If the reliability score is the best possible for the type of structure being processed (*viz.* 3 for uncharged organics, 2 for everything else, see later), the assignment is accepted and the iterative process terminated. Otherwise, it is stored in case no better can be found, bond types and atom charges set back to unknown and cycle 2 of the iteration begun. The most probable of the bond type options not chosen at the start of cycle 1 is identified and used to replace whichever bond-type option was previously chosen for the UF to which it belongs. The procedure is then as in cycle 1. Iteration is terminated when a sufficiently reliable assignment is found or when the maximum number of iterations is reached (we allow up to 10 cycles). If this happens, the Mogul algorithm is tried on its own (*i.e.* no use of UFs) in case it can find a solution of higher reliability.

### 5.9. Assignment of charges and missing H atoms   

Once a trial bond-type assignment has been made, it is necessary to assign charges and deduce whether any H atoms are missing. For each atom in the structure (excluding H atoms), one UF is chosen, if possible, as the best source of information about the likely charge and hydrogen-count of that atom. Complete-molecule UFs are regarded as the most reliable source, followed by complete-ligand, substructure, and individual-atom UFs. In MIJFOA the chosen UFs include all the complete-molecule and complete-ligand UFs. Bond types having been assigned, one bond-type option will have already been selected for each of these UFs. The probabilities of all atom-property options associated with the chosen bond-type options are calculated (as described in §5.8).

The procedure is then analogous to the iterative method described above for bond-typing. In the case of MIJFOA, the outcome is that three missing H atoms are inferred on the terminal acetonitrile C atom, together with the negative charge on the tetrafluoroborate. If the choice of atom-property options does not lead to a zero net charge over the unit cell, an attempt is made to rectify the problem by assigning charges to any metal atoms that might be available (hence, in MIJFOA, a balancing +1 charge is placed on the Cu atom). If this is unsuccessful (*i.e.* charges cannot be balanced), the trial structure assignment is obviously incorrect and is awarded the lowest possible reliability score. Where relevant, it is usually desirable and often essential to distribute charges equally over topologically equivalent atoms and molecules. For example, if two identical molecules in the asymmetric unit need to accommodate, between them, a charge of +2, it is usually better to make each carry a charge of +1 rather than have one neutral and one di-cationic. Further, if there is a choice of atoms on which to place the charge, it is desirable (albeit sometimes for cosmetic reasons) to choose the same atom in each molecule on which to place the charge.

### 5.10. Miscellaneous details   

The assignment may be run in two modes:

(i) assuming that there are no missing H atoms (in which case atom-property options that indicate missing H atoms are only accepted as a last resort), and
(ii) considering that missing H atoms are likely. We use both modes in turn and take the best answer.

At the end of the procedure described in §5.8, all metal–metal bond orders will have been assigned on the basis of individual-bond UFs, using geometry-biased probabilities from (3). The correlation between metal–metal bond order and bond length is rather poor so, at the end of the assignment, each metal–metal bond is re-examined. If the probability of the assigned bond type minus that of the next most probable is ≥ 0.3, it is accepted. Otherwise, the bond order is determined by counting metal valence electrons, assuming the remainder of the structure assignment is correct. If the electron counting indicates a different bond type from that assigned, the revised value is accepted unless its probability, as calculated from the individual-bond UF, is low (< 0.075). Non-integral bond orders obtained by electron counting are approximated by the more probable of the bracketing, integral bond types.

## 6. Assessment of structure-assignment reliability   

Each assigned structure is given a *reliability score* which can take the values 0, 1, 2 or 3, larger values indicating greater reliability. The assessment procedure is rule-based and was developed empirically. Table 2 ▸ lists the dependence of the score on the various assessment criteria used. These fall into two categories. Some, such as the presence of a metal atom, are not in themselves indicative of error but are features known to make structure assignment difficult and therefore less reliable. Others, such as a non-planar double bond, are directly suggestive of possible error. In addition to the score, warning messages about suspect features are reported. Table 3 ▸ shows an example for a structure assignment of relatively low reliability. Reports such as this often indicate clearly the points of error in the assigned structure.

**Figure 13:**
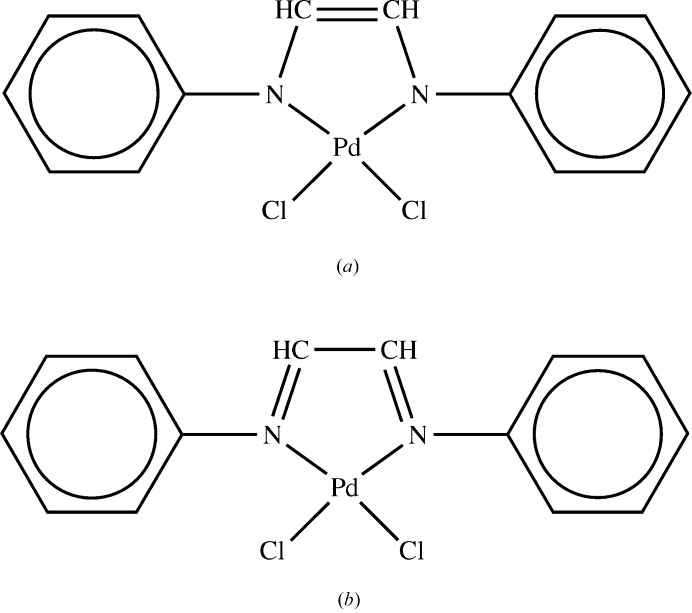
Two representations of a palladium complex implying different metal oxidation states.

Of particular importance is the low-probability oxidation state. With a few exceptions (*e.g.* metal atoms in clusters) the template procedure of Shields *et al.* (2000 ▸) is used to calculate the oxidation state implied for each metal atom by the structure assignment that has been made. For example, the method would infer an oxidation state of +4 for the Pd atom of the structure shown in Fig. 13 ▸(*a*), since each ligand N atom has a notional charge of −1, but will give +2 for the alternative assignment in Fig. 13 ▸(*b*), where each nitrogen is notionally uncharged. Since Pd^II^ is much more common than Pd^IV^, this would lower the reliability score of the former assignment. This type of evidence is often the clearest indication of assignment error. Where possible, metal-atom oxidation states are also estimated by the bond-valence sum (BVS) method (Brown, 2002 ▸), which gives values based on the geometry around the metal atom (*i.e.* the estimates are independent of the assigned bond types and atom charges). A discrepancy between template and BVS estimates is suggestive of possible error.

When calculating oxidation states for polymers, there is the danger of ‘edge effects’ because metal-coordination spheres near polymeric linkages may be incomplete. To avoid this, the calculation is carried out on the multimer obtained by adding an extra unit at each polymeric bond (*e.g.* for a linear polymer with representative unit *M*, the calculation is done on *M*–*M*–*M*).

## 7. Illustrative results   

This section describes illustrative results based on the CSD entries discussed in §1 (Figs. 1–7 ▸ ▸ ▸ ▸ ▸ ▸ ▸), starting with GEBXOA. This is assigned with a triple bond between the Ru atoms. The actual bond order from electron counting is 2.5 (Chakravarty *et al.*, 1986 ▸). Metal–metal multiple bonds are often assigned correctly, although it is also common for the assigned bond order to be out by 1 in either direction. Missing H atoms in GEBXOA are inferred correctly.

The charges on the metal-containing species in HEWMOL are assigned correctly, the algorithm recognizing that the implied metal oxidation states – Mn^II^, Tl^I^ and Tl^III^ – are all of high probability. The assignment is easy because the MnCl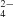 ion is in the complete-molecule UF datafile, and therefore known to be invariably di-anionic. Also, thallium has very well defined oxidation-state preferences. The algorithm is often successful in such circumstances. For example, zinc complexes are usually assigned correctly because the metal can only be Zn^II^; even when errors are made, the oxidation-state check usually indicates that there is a problem. Conversely, structures containing two or more metals which can adopt many oxidation states are much more likely to be assigned incorrectly (although often with low reliability scores), especially when, as is common, none of the metal-containing molecules or ions correspond to entries in the complete-molecule UF data file.

The assignment of HEWMOL is not perfect because the algorithm does not identify bonds between the Tl^+^ ion and the crown ether O atoms (these bonds are present in the CSD representation). This is a common situation in highly ionic complexes (most obviously, when oxygen ligands are coordinated to elements of groups 1 and 2), where the distinction between a metal–oxygen bond and a metal⋯oxygen short nonbonded contact is blurred. It is then difficult for the algorithm to reproduce what is essentially the subjective judgement of a chemist. The identification of metal–oxygen bonds in these types of compounds is an ongoing problem in the CSD. The policy of following authors’ judgements leads to inconsistencies, which places an onus on database users to construct substructure queries with care. Conversely, if bonds were assigned on the basis of strict distance criteria, the result in many cases would be chemically unintuitive.

YAZZOP and BALTUE are assigned correctly, with the redox-active ligand in its correct oxidation state in each. However, these types of structures represent a severe challenge for the algorithm and errors are frequent, although they are often highlighted by oxidation-state warnings. The correct assignment of Re=O double bonds in BALTUE is satisfying given the superficially attractive alternative of assuming the O atoms belong to water molecules with undetermined H-atom positions. Metal–oxygen and metal–nitrogen double bonds are common so it is important to recognize them, and the algorithm tends to perform well in this respect. Bond-length differences between single and double bonds can be substantial (*e.g.* about 0.4 Å between the mean values of V—OH_2_ and V=O), which helps.

The algorithm fails to reproduce the CSD charge assignment for BAPYEX, making all species neutral and compounding the felony by awarding a relatively high reliability score of 2. Unfortunately, this is typical: the algorithm performs badly on charge-transfer salts. The authors describe BAPYEX as a biradical (Mochida *et al.*, 2002 ▸), which cannot be properly represented in the CSD anyway.

VOMNUH and VOMPAP are both assigned correctly with reliability scores of 2. The algorithm assigns an aromatic representation to the pyrazole ligands in VOMNUH, since both N atoms are bonded to metal and the negative charge is therefore unlikely to be localized on either one of them. An aromatic representation would also be assigned to a pyrazole ligand in which one N atom was metal-bound and the other bonded to boron, since boron is a metalloid. However, if one of the N atoms is bonded to a metal and the other to a non-metal, as in VOMPAP, a non-aromatic representation results.

The carbene ligands in XONQIB are correctly identified as such (this is often but not always the case). However, the assignment differs from that in the CSD in that the CN bonds in the carbene ligand are assigned as single rather than delocalized. In our view, either representation is defensible.

OFIKOD (Fig. 2 ▸) is assigned incorrectly because the missing metal-bound hydrogen is not inferred. However, the incorrect structure is accompanied by warning messages and awarded a reliability score of only 1 (this is the structure to which Table 3 ▸ refers). The template-based oxidation-state method detects that the assigned structure implies Pt^I^, which has low probability (*p* = 0.009). This is a typical result: missing metal-bound H atoms are never added by the algorithm but the error often produces oxidation-state warnings. In the present case, there is an additional clue: without the hydride ligand, the Pt atom appears to be three-coordinate with an unusual ‘T’-shaped geometry. However, the algorithm currently makes no use of metal coordination geometries.

XOLSIB and VOLSAR (Fig. 3 ▸; both assigned correctly) represent another common and difficult problem on which the algorithm often fails: whether to assume a metal-bound —O*R* group is an alkoxide or an alcohol with a H atom missing. Again, the oxidation state is usually the biggest clue. In XOLSIB the assumption of missing alcohol H atoms is necessary to achieve a template-based oxidation state estimate of Dy^III^, which is the only reasonable hypothesis for this element. Conversely, a credible oxidation state is obtained for the Nb atom in VOLSAR with the alkoxide formulation, which is therefore accepted. In general, the algorithm tends to avoid inferring missing H atoms unless their presence is very obvious.

NOLZOE (Fig. 4 ▸) is assigned correctly: in particular, the solvate molecule is assigned as tetrahydrofuran despite its near planarity. This reflects the influence of the prior probabilities in Bayes’ formula, tetrahydrofuran occurring in the CSD several hundred times more often than furan. Interestingly (and somewhat to our surprise) the few furan molecules in the CSD are often assigned correctly, *e.g.* in CSD entries GAGBEV and WOSREB, suggesting that the geometry tests are well chosen for the relevant complete-molecule UF. In contrast, cyclohexane molecules with missing H atoms are almost always assigned as the overwhelmingly more common benzene, suggesting that the geometry tests are less effective for this pair. Cyclohexane geometries in the CSD are very variable (*i.e.* parameters used in geometry tests have large standard deviations), and we suspect this reduces the discriminatory power of the tests.

As mentioned earlier, the algorithm does not resolve the disorder in QEHLOF (Fig. 5 ▸), where H atoms are present only for the major configuration of a disorder assembly. This situation is typical of cases where it is probably better to rely on manual editing than attempt an algorithmic solution. In DEHMAF (Fig. 6 ▸), the algorithm correctly assumes that the structure contains methanol disordered by symmetry over two sites rather than half-occupancy ethane-1,2-diol. However, it will always make this type of assumption. In another example, TOLLOW, this gives the wrong answer – the structure is supposed to contain partial occupancy NH_2_—CH_2_—CH_2_—NH_2_, but the algorithm assumes disordered CH_3_NH_2_. There are two H atoms on each solvent carbon in this structure, which suggests the authors intended the former description, but not conclusively (*i.e.* one of the H atoms on each carbon might have been missing).

The symmetry-imposed disorder in AHALEA (Fig. 7 ▸) is resolved correctly. The twelve 1/4 occupancy oxygen sites (generated from three symmetry-independent oxygen sites by a fourfold axis) are correctly partitioned into four groups, each representing a reasonable sulfate-ion geometry, thus demonstrating that the geometry-scoring function is effective.

## 8. Algorithm validation   

The algorithm was validated on a random sample of 1777 structures with CSD accession dates falling in May 2009. None of the structures was used in developing the algorithm or contributed to its underlying data files. The CIFs received by the CCDC were used as input and the resulting structure assignments were compared with those in the CSD, all of which were created by the normal CCDC editing process. Each algorithmically produced assignment was categorized as *identical*, *acceptable* or *incorrect*. Identical assignments were those for which there was an exact match (bonds, bond types, atom charges, inferred missing H atoms, and, where relevant, polymer unit) with the corresponding CSD assignment for all molecules and ions in the asymmetric unit, including disordered moieties.

An algorithmically derived assignment was categorized as *acceptable* if all differences between it and the CSD version were either trivial or at least chemically defensible and unlikely to mislead a CSD user. The types of discrepancy most often leading to this classification were, in descending order of frequency, as follows.

(i) Differences in polymer representation. (To the best of our knowledge, the algorithm always picks a polymer unit with correct metal:ligand stoichiometry but it often differs from that chosen by the CSD editors. Frequently, the algorithm selects a multimer where editors prefer a monomer.)
(ii) Minor differences in the use of single, double and delocalized bonds, *e.g.* the bonds of a bridging phosphonate group might be represented as delocalized or single and double.
(iii) Differences arising from marginal decisions on the existence of metal–non-metal bonds, *e.g.* representation of an Sn atom as five-coordinate with one long Sn—S bond or as four-coordinate with an additional short Sn⋯S non-bonded contact.
(iv) Trivial differences in charge placement, *e.g.* negative charges placed on different O atoms in a Keggin structure.
(v) Different but equally defensible representations of carbene ligands (*c.f.* XONQIB, Fig. 1 ▸ *f*, discussed above).

Table 4 ▸ summarizes the numbers and percentages of identical, acceptable and incorrect assignments, broken down by assignment reliability level. The overall success rate (counting success as either *identical* or *acceptable*) is 73.8%, rising to 87.7 and 98.0% for assignments with reliability levels 2 and 3. Assignments with reliability level 0 are usually incorrect. All incorrect assignments were inspected visually to identify the cause(s) of failure. Results are summarized in Table 5 ▸. The most common problem is failure of the bond type and/or charge-assignment algorithm, followed by problems of disorder resolution and then failures in the bond-detection algorithm. A significant minority of failures result from incomplete information in the incoming CIF. Finally, a handful of errors arose from technical bugs in the program.

A more detailed analysis showed the following to be the major causes of incorrect structure assignment, discussed in descending order of importance:

(i) Failure to resolve solvent disorder (occurring in about 60 structures). The disorder is very often symmetry imposed and/or severe. Frequently, the remainder of the structure is assigned correctly.
(ii) Discrepancies in whether metal–metal bonds are deemed to exist (about 57 examples). While the algorithm uses electron counting to check and, if necessary, amend the assigned order of a metal–metal bond, it is not used to help decide on the existence of the bond in the first place; this is currently based only on distance considerations.
(iii) Missing solvent atoms, including the modelling of solvent (and occasionally small ions) by the SQUEEZE option of *PLATON* (Spek, 2005 ▸; about 48 examples). In these cases, the atom coordinates necessary for correct assignment are not present in the CIF.
(iv) Failure to infer (or, less commonly, incorrect inference of) the presence of missing H atoms (about 45 examples). The H atoms in question are invariably either metal-bound hydrides or atoms attached to ionisable groups.
(v) Incorrect bond typing of unsaturated systems, including confusion between single/double and aromatic representations, *e.g.* for redox-active ligands (about 45 examples).
(vi) Discrepancies in whether metal–non-metal bonds are deemed to exist, including differences in hapticity (about 37 examples).
(vii) Failure to suppress disordered atoms (about 30 examples). CSD conventions dictate that a disordered group is represented by its major configuration, all others being ‘suppressed’ (see above). Due to a programming oversight, the algorithm occasionally resolved disorder correctly but omitted the suppression step.
(viii) Failure to resolve (non-solvent) symmetry-imposed disorder (about 21 examples).
(ix) Incorrect representation of an ion pair as two neutral molecules, *c.f.* BAPYEX, discussed above (about 17 examples).

The test set contains 671 structures that are classified in the CSD as organic, but 49 of these contain group 1 or 2 metal ions, some of which form pi or metal–metal bonds. The success rate for the remaining 622 structures is 87.6%, rising to 98.0% (93.3%) for the 406 (104) structures with reliability level 3 (2). Of the 77 errors, almost half (36) are due to disorder. Failure to perceive aromaticity, especially in sulfur–nitrogen rings, is the next most common problem (14 errors), followed by incorrect charge placement (nine errors), and problems due to severe errors in the CIF (six errors). The remaining errors are due to a variety of causes, including unusual bonding situations (*e.g.* multiple silicon–silicon bonds).

## 9. Conclusions   

We have described the structure-assignment algorithm used to help CCDC editors add new entries to the CSD. Effectively, the algorithm exploits the chemical information in the CSD to interpret and add value to the atomic coordinates obtained from the diffraction experiment. The algorithm has the potential for wider use as a tool for adding chemical knowledge to newly determined crystal structures, thereby increasing the degree to which high-throughput crystallography can be automated. It has also facilitated the release of entries as part of the CSD X-Press system. Entries in CSD X-Press have had chemistry assigned by the new structure-assignment algorithm and are accompanied by an automatically generated two-dimensional diagram, together with data items that are available in the CIF. When no compound name is present in a CIF, an attempt is made to automatically generate one based on the assigned chemistry using ACD/Name (Advanced Chemistry Development, Inc., 2010 ▸). Importantly, entries in CSD X-Press are given a star rating based on the reliability score produced by the structure-assignment algorithm. This provides users with an indication of the confidence they can have in the chemical assignment when deciding how to handle structures as part of a scientific study. CSD X-Press entries are made available through WebCSD (Thomas *et al.*, 2010 ▸) where they are clearly highlighted as pending enhancement (*e.g.* resolution of any structure-assignment problems) by editorial staff before inclusion in the main CSD. The introduction of CSD X-Press allows earlier public release of structures that have value added to the data present in the original CIFs, primarily as a result of the new structure assignment algorithm.

At first sight, the overall success rate of the algorithm (∼ 74%) may appear poor compared with those reported for published bond-type assignment methods intended primarily for use on PDB ligands, which typically achieve success rates of 90 to 95%. However, CSD structure assignment is a different proposition. First, our starting point is not a discrete molecule but the raw CIF received by the CCDC, the information in which may be incomplete (*e.g.* through the use of the SQUEEZE algorithm). Second, our success rate is measured on a per-structure rather than a per-molecule basis, *i.e.* all molecules in the asymmetric unit, including any solvent that might be present, must be set correctly. Third, we attempt to resolve disorder, including symmetry-imposed disorder, which is almost never a problem in macromolecular structures. Fourth, we use an unusually extensive set of bond types. Fifth, and critically, our validation set contains a large number of metallo-organic complexes and we require that assigned structures correctly reflect authors’ interpretations of ionic charges, metal oxidation states, hapticity, *etc*.

Since a 100% success rate is unrealistic, the most important feature of the algorithm is that it generates meaningful estimates of reliability and, when in error, produces diagnostic information that indicates the dubious aspect(s) of the assignment (most erroneous assignments are incorrect only at one or two points in the structure). These reliability estimates highlight those entries most in need of attention by CSD editors. The role of manual editing continues to be critical, not only because of the deficiencies in the algorithm, but also to maintain a solid foundation of highly reliable structure assignments that can be used as the basis for future versions of the untyped-fragment data files on which the algorithm depends.

While the algorithm is useful as it stands, there is need for further improvement. The most important figures in Table 4 ▸ are the failure rates for structures whose reliability levels are 3 or 2, *viz.* 2.0 and 12.3%. Arguably, the former is sufficiently small that structures with reliability level 3 could, if necessary, be added to the CSD with no editorial inspection. The latter is not. If we are to attain a position in which the majority of assigned structures can be accepted without manual inspection, we therefore need to improve the success rate for structures with reliability level 2. Alternatively, we could make the assessment of reliability more discriminating so that some of the correctly assigned structures at level 2 are moved to level 3.

Some of the major causes of structure-assignment error are rather intractable. They include the inference of some types of missing H atoms, assignment of charges in charge-transfer structures, assignment of redox-active ligands coordinated to metals that can adopt several oxidation states, and severe (especially symmetry-imposed) disorder. Mixed-element disorder and the related problem of misassignment of atom types (*e.g.* nitrogen for carbon) is particularly difficult to resolve. Frequency-of-occurrence data can help identify possible problems (*e.g.* nitriles are much more common than isonitriles), but will not resolve them unambiguously.

Other problem areas may be easier to address. The issue of unsuppressed disordered atoms [see (vii)[Other l8li7] in the preceding section] has already been fixed since the validation was performed. Some types of disorder might be better resolved by the use of solvent frequency data. For example, the information that methanol is much more common in the CSD than ethylene glycol could have been used to resolve the disorder in DEHMAF (§7) with greater confidence. It should be possible to reduce the error rate in assigning metal–metal bonds by an additional electron-counting step after an initial structure assignment. Use of the 18-electron rule and Wade’s rules would be valuable for checking the structure assignments of metal complexes such as cluster carbonyls, where oxidation state is not a particularly meaningful concept. Molecules modelled by the SQUEEZE algorithm might be identified by parsing CIFs for common solvent names. Data on common metal coordination geometries might be used to improve the accuracy of metal–non-metal bond detection and the inference of missing metal hydrides. It may be possible to identify algorithmically the most discriminating geometry tests for distinguishing bond-type and atom-property options. Analysis of intermolecular contacts may aid the inference of missing H atoms and thus help distinguish, for example, alkoxide from alcohol ligands. The algorithm uses several empirically chosen parameters that could be more thoroughly optimized. Perhaps most important of all, the algorithm often detects errors but is unable to fix them. This suggests that a better search algorithm should be implemented, which in turn would require a more sensitive scoring function than the simple reliability level used at present.

Finally, our work emphasizes the importance of carefully preparing a detailed CIF. No one enjoys this chore, but it produces tangible benefits for the crystallographic community and will become increasingly important as the productivity of crystallographers continues to rise.

## Figures and Tables

**Table 1:** Statistics for complete-molecule untyped fragment CCN S.d. = standard deviation (default values used for CH_3_CH_2_NH_2_).

Bond-type option		CCN	CCN	
Atom-property option		CH_3_CN	CH_3_CH_2_NH 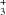	CH_3_CH_2_NH_2_
No. observations		5102	40	4
CCN angle ()	Mean	175.02	113.76	113.05
	S.d.	8.23	6.20	4.00
CC distance ()	Mean	1.432	1.473	1.497
	S.d.	0.082	0.120	0.040
CN distance ()	Mean	1.142	1.488	1.505
	S.d.	0.080	0.060	0.040

**Table 2:** Criteria used to set structure-assignment reliability score

Score = 0 if any of:
*Unbalanced charges*: charges on atoms do not sum to zero over unit cell.
*Unprecedented atoms*: atom has assignment never observed in CSD (unknown atom-property option).
*Unprecedented bonds*: bond has type never observed in CSD (unknown bond-type option).
*Double-bonded C(ar)*: C atom forms both aromatic and double bonds.

Score = 1 if none of the above and any of:
*Low-probability atoms*: an accepted atom-property option has probability 0.01.
*Low-probability bonds*: an accepted bond-type option has probability 0.01 (excluding bonds in common solvate molecules).
*Discrepant metalmetal bond order*: assigned metalmetal bond order differs from that calculated by electron counting by > 0.5.
*Low probability oxidation state*: template-method gives oxidation state with probability 0.1 for at least one metal atom.
*Oxidation-state discrepancy*: for at least one metal atom, template and BVS methods give oxidation states differing by > 0.99 (test omitted if BVS estimate is of low probability).
*Oxidation state could not be calculated*: oxidation state of at least one metal atom could not be calculated by template method (*e.g.* because of missing template).
*(X)H missing from large molecule*: assignment indicates at least one (*X*)H atom (*X* C) missing from a molecule with > 9 non-H atoms (or any molecule if all in unit cell have 18 non-H atoms).
*H atom added to planar atom*: assignment requires an H atom to be added to an atom already bonded to three atoms in a trigonal geometry.
*H atom added to linear atom*: assignment requires an H atom to be added to an atom already bonded to 2 atoms in a linear geometry.
*Group 1 or 2 metal present.*

Score = 2 if none of the above and any of:
*Non-planar double or aromatic bond*: torsion angle > 20 from planarity.
*Difficult substructure present*: *e.g.* tetrathiafulvalene (forms charge-transfer complexes), metal-bound alkoxide (protonation state difficult to determine).
*(C)H missing from large molecule*: assignment indicates at least one (C)H atom missing from a molecule with > 9 non-H atoms (or any molecule if all in unit cell have 18 non-H atoms).
*Charges assigned*: two or more atoms assigned non-zero charges.
*Metal present.*

Score = 3 if none of the above.

**Table 3:** Typical diagnostic report from structure-assignment program

Oxidation states (template method):
Pt1 1, Pt2 1
Oxidation states (BVS method):
Pt1 2.0, Pt2 2.0
Low probability oxidation states:
Pt2, prob = 0.009
Pt1, prob = 0.009
Electron counts (template method):
Pt1 15, Pt2 15
Low probability bond lengths:
F14C58 1.515, av(CSD) = 1.319, prob = 0.001
F10C57 1.673, av(CSD) = 1.319, prob = 0.001
C57C56 1.603, av(CSD) = 1.527, prob = 0.002
Reliability level = 1

**Table 4:** Validation results: numbers and percentages of identical, acceptable and incorrect structure assignments, broken down by reliability level

	Counts (percentages)			
Reliability level	Total	Identical	Acceptable	Incorrect
3	408	398 (97.5%)	2 (0.5%)	8 (2.0%)
2	733	531 (72.4%)	112 (15.3%)	90 (12.3%)
1	425	124 (29.2%)	97 (22.8%)	204 (48.0%)
0	211	36 (17.1%)	11 (5.2%)	164 (77.7%)
All	1777	1089 (61.3%)	222 (12.5%)	466 (26.2%)

See text for definition of identical, acceptable, incorrect.

**Table 5:** Causes of incorrect assignments, broken down by reliability level

	Number of incorrect assignments caused by failures in				
Reliability level	CIF	Disorder resolution	Bond detection	Bond types, atom charges	Bug
3	3	3	0	2	0
2	22	24	29	17	3
1	29	24	48	113	5
0	9	87	28	50	2
All	63	138	105	182	10

CIF = incomplete or grossly inaccurate information in the CIF.

Some incorrect assignments were ascribed to more than one cause.
